# Childhood maltreatment trauma: a comparison between patients in treatment for substance use disorders and patients in mental health treatment

**DOI:** 10.1080/20008198.2018.1492835

**Published:** 2018-07-17

**Authors:** Ingvild S. Rasmussen, Kjersti Arefjord, Dagfinn Winje, Anders Dovran

**Affiliations:** a Outpatient Department, Karmøy District Psychiatric Center, Kopervik, Norway; b Faculty of Psychology, Department of Clinical Psychology, University of Bergen, Bergen, Norway; c The Stine Sofies Foundation and The Stine Sofie Centre, Grimstad, Norway

**Keywords:** Child abuse, neglect, post-traumatic stress symptoms, Childhood Trauma Questionnaire, avoidance symptoms, self-medication model, dual diagnosis, Abuso infantil, negligencia, síntomas de estrés postraumático, Cuestionario de Traumas de la Infancia, síntomas evitativos, modelo de auto-medicación, diagnóstico dual, 儿童虐待, 忽视, 创伤后应激症状, 童年创伤问卷, 回避症状, 自我治疗模型, 双重诊断, • In the present study, patients in treatment for substance use disorder reported a history of more various and severe childhood maltreatment trauma compared to mental health patients without substance abuse.• Females in treatment for substance use disorder reported a history of more various and severe childhood maltreatment trauma compared to males.• The patients in treatment for substance use disorder reported more avoidance symptoms compared to patients in psychological treatment without substance use disorder.

## Abstract

**Background:** While previous research has found strong associations between childhood maltreatment trauma and substance use disorders (SUDs), the role of possible moderating effects of gender and mediating effects of psychopathology and SUD is unclear.

**Objective:** The objective of this study was to investigate differences in self-reported childhood maltreatment trauma, general psychological distress, and post-traumatic stress symptoms between 112 patients in treatment for substance use disorders (SUD group) and 112 matched controls with mild to moderate mental health disorders (comparison group).

**Methods:** Childhood maltreatment trauma was measured by the Childhood Trauma Questionnaire – Short Form (CTQ-SF). General psychological distress was measured by the Symptom Checklist-90 – Revised (SCL-90-R), and post-traumatic stress symptoms were measured by the Impact of Event Scale – Revised (IES-R).

**Results:** The SUD group reported more severe childhood maltreatment trauma than the comparison group. Females in the SUD group reported more severe and various forms of trauma compared to males. The SUD group reported higher mean scores on the SCL-90-R, but the proportions of people with caseness scores on the IES-R and the SCL-90-R were similar in the two samples. The SUD group reported more avoidance symptoms than the comparison group.

**Conclusion:** This study adds further evidence to the repeatedly found strong associations between childhood maltreatment trauma and SUD, implying that the prevention of childhood maltreatment trauma may reduce the occurrence of SUD. Furthermore, patients with SUD should be screened for childhood maltreatment trauma, and the results should be applied in trauma-informed as well as trauma-focused interventions aimed to help this population. The association appears to be particularly strong for female substance users.

## Abstract (es)

**Antecedentes:** Si bien investigaciones previas han encontrado fuertes asociaciones entre el trauma por maltrato infantil y los trastornos por uso de sustancias (SUD), no está claro el papel de los posibles efectos moderadores del género y los efectos mediadores de la psicopatología y el SUD.

**Objetivo:** El objetivo de este estudio fue investigar las diferencias en el trauma de maltrato infantil autoreportado, el estrés psicológico general y los síntomas de estrés postraumático entre 112 pacientes en tratamiento por trastornos por consumo de sustancias (grupo SUD) y 112 controles emparejados con trastornos de salud mental leves a moderados (grupo de comparación).

**Métodos:** el trauma por maltrato infantil se midió mediante el Cuestionario de Traumas de la Infancia, versión abreviada (CTQ-SF). El estrés psicológico general se midió mediante la Lista de Chequeo de Síntomas Revisada (SCL-90-R), y los síntomas de estrés postraumático se midieron mediante la Escala de Impacto de Eventos Revisada (IES-R).

**Resultados:** El grupo SUD reportó trauma de maltrato infantil más severo que el grupo de comparación. Las mujeres en el grupo SUD reportaron formas más graves y diversas de trauma en comparación con los hombres. El grupo SUD informó puntuaciones medias más altas en el SCL-90-R, pero las proporciones de personas con puntajes de casos en el IES-R y el SCL-90-R fueron similares en las dos muestras. El grupo SUD informó más síntomas de evitación que el grupo de comparación.

**Conclusión:** Este estudio agrega evidencia adicional a las fuertes asociaciones encontradas repetidamente entre el trauma por maltrato infantil y el SUD, lo que implica que la prevención del trauma de maltrato infantil puede reducir la ocurrencia de trastornos por uso de sustancias. Además, los pacientes con SUD deberían ser evaluados para detectar traumas por maltrato infantil y los resultados deberían aplicarse en intervenciones basadas en el trauma e intervenciones focalizadas en el trauma dirigidas a ayudar a esta población. La asociación parece ser particularmente fuerte para las mujeres usuarias de sustancias.

## Abstract (zh)

**背景：**虽然以前的研究发现童年虐待创伤和物质滥用障碍（SUD）之间有强关联，但性别的调节效应和精神病理的中介效应对SUD的作用尚不清楚。

**目的：**本研究的目的是比较112例物质滥用障碍治疗组（SUD组）和112例与其匹配控制的轻度至中度精神健康障碍患者（比较组）的自我报告童年虐待创伤、一般心理困扰和创伤后应激症状的差异。

**方法：**使用《童年创伤问卷简表》（CTQ-SF）测量童年虐待创伤，《症状清单-90-修订版》（SCL-90-R）测量一般心理困扰，并且使用《事件影响量表修订版》（IES-R）来测量创伤后应激症状。

**结果：**SUD组比对照组报告更严重的儿童虐待创伤。与男性相比，SUD组中的女性报告了更严重和更多形式的创伤。 SUD组报告SCL-90-R的平均得分较高，但IES-R和SCL-90-R的病例比例在两个样本中相似。 SUD组报告比对照组更多的回避症状。

**结论：**这项研究进一步证实了童年虐待创伤与SUD之间反复发现的强关联，这意味着预防儿童虐待创伤可能会减少SUD的发生。并且，患有SUD的患者应该接受儿童虐待创伤的筛查，并将结果应用于创伤相关和创伤中心的干预措施，以更好帮助这些人群。这种关联性似乎在女性物质滥用者中变现更强。

## 1. Introduction

The World Health Organization (WHO) has defined the term *childhood maltreatment* as ‘all forms of physical and emotional ill-treatment, sexual abuse, neglect, and exploitation that results in actual or potential harm to the child’s health, development or dignity’ (World Health Organization [WHO], 2016). Childhood maltreatment is common, with millions of children affected every year (Butchart, Harvey, Mian, & Furniss, 2006). For many, it is a chronic and often undiscovered condition (Gilbert et al., 2009). A substantial amount of research suggests that childhood maltreatment trauma (CMT) is closely connected to adult mental health problems, and it has been estimated that adverse childhood experiences can be directly linked to approximately 30% of all mental health disorders (Kessler et al., 2010) and to 50–66% of serious drug abuse (Dube et al., 2003).

The association between CMT and later substance use disorders (SUDs) appears to be strong, with more severe maltreatment increasing the association (Douglas et al., 2010; Dube et al., 2003). Previous research has found experiences of CMT to be associated with a younger age at initiation to substance use (Tonmyr, Thornton, Draca, & Wekerle, 2010) and more serious substance abuse (Taplin, Saddichha, Li, & Krausz, 2014). The association between CMT and SUD appears to be particularly strong for females (Gilbert et al., 2009), and females show increased vulnerability to the adverse consequences of SUD (Back, Contini & Brady, 2007).

Several emotional disorders are associated with substance abuse, and mental health problems may mediate the association between CMT and SUD (Simpson & Miller, 2002). In a study of young adults, Reed, Anthony, and Breslau (2007) found that traumatic experiences were associated with excess risk for substance abuse or dependence only if mediated by the occurrence of post-traumatic stress disorder (PTSD). A substantial amount of research has confirmed a close relation between PTSD and SUD, suggesting that 30–60% of individuals seeking help for SUD meet the criteria for lifetime PTSD (Brady, McCauley, & Back, 2015). However, Gielen, Havermans, Tekelenburg, and Jansen (2012) found that SUD patients with PTSD were more likely to have additional depressive or other axis I disorders compared to SUD patients without PTSD, suggesting that comorbid psychological disorders are frequent among traumatized substance abusers.

Research indicates that individuals who have experienced sustained, repeated or multiple traumas in childhood develop a complex form of PTSD (Herman, 1992; van der Kolk, Roth, Pelcovitz, Sunday, & Spinazzola, 2005). These patients often meet the diagnostic criteria for comorbid psychiatric diagnoses (Cloitre et al., 2009), with disturbances in emotion regulation, self-concept, and relational skills (Cloitre, Garvert, Brewin, Bryant, & Maercker, 2013). Neurological studies have confirmed an association between CMT and maturation failures in the frontal and prefrontal cortex that negatively affect executive functioning and self-regulation skills, in addition to alterations in the major stress-response systems (De Bellis, 2002).

CMTs associated with maladaptive family function and interpersonal trauma are linked to the greatest risk of mental health disorders in adult life (Alisic et al., 2014; Green et al., 2010; Kessler et al., 2010). It is estimated that at least 80% of childhood maltreatment is perpetrated by parents or legal guardians, except for childhood sexual abuse, which is mostly perpetuated by other relatives or acquaintances (Gilbert et al., 2009). Multiple childhood adversities appear to be common among individuals who report any such experiences (e.g. Dong et al., 2004; Dube et al., 2003; Green et al., 2010; Kessler et al., 2010), and exposure to multiple traumas in childhood is associated with increased symptom complexity in children and adults (Cloitre et al., 2009; Finkelhor, Ormrod, & Turner, 2007). Although many earlier studies focused on finding specific outcomes related to specific forms of maltreatment, later studies found no such associations (Green et al., 2010; Kessler et al., 2010; Vachon, Krueger, Rogosch, & Cicchetti, 2015).

The present study examined the prevalence of five types of CMT – emotional abuse, physical abuse, sexual abuse, emotional neglect, and physical neglect – and how they correlated with current post-traumatic stress symptoms and general psychological distress in matched control samples of adults in treatment for SUD and adults without SUD in treatment for mental health problems. We hypothesized the following: (1) the patients with SUD would report more CMT than the comparison group; (2) within the SUD group, the females would report more CMT than the males; (3) reports of CMT would be associated with symptoms of general psychological distress and current post-traumatic stress symptoms; and (4) multiple traumas would be common among patients who reported a history of any trauma.

## 2. Methods

### 2.1. Participants

The data were collected by the Trauma Psychology Research Group, University of Bergen. The participants consisted of 121 patients diagnosed with SUD and 121 matched controls with mild to moderate mental health disorders without SUD. Nine matched pairs were excluded from the study owing to missing consent, problematic substance use in the comparison group or missing data on the Childhood Trauma Questionnaire – Short Form (CTQ-SF). No differences were found in age or Symptom Checklist-90 – Revised Global Severity Index (SCL-90-R GSI) scores between the excluded patients and the rest of the sample. We were left with 112 matched pairs, each group consisting of 63 males and 49 females between the ages of 17 and 63 years (Table 1).

**Table 1.** Demographic characteristics by group (*N = *112 for both groups).

	SUD group (*n* = 112)	Comparison group (*n *= 112)
Age (years)	27.5 (8.3)	27.5 (8.3)
Gender, male	63 (56.3)	63 (56.3)
Marital status		
Single	80 (71.4)	66 (58.9)
Married or living with partner	22 (19.6)	43 (38.4)
Divorced or widowed	9 (8.0)	2 (1.8)
Unknown	1 (0.9)	1 (0.9)
Education		
Primary school or less education	51 (45.5)	13 (11.6)
College or higher education	60 (53.6)	99 (88.4)
Unknown	1 (0.9)	0 (0)
Living situation		
Own home or living with others	103 (92.0)	111 (99.1)
Institutionalized or homeless	8 (7.1)	0 (0)
Unknown	1 (0.9)	1 (0.9)
Work status		
Working and/or studying	56 (50.0)	100 (89.3)
Neither working nor studying	55 (49.1)	12 (10.7)
Unknown	1 (0.9)	0 (0)

Data are shown as *n* (%).

SUD, substance use disorder.

The SUD group was derived from four different inpatient and outpatient treatment clinics in the area of Bergen, Norway (Bergen Clinics Foundation: Clinical Department Hjellestad; Psychiatric Youth Team, Department of Addiction Medicine, Haukeland University Hospital; Askøy Treatment Centre, Department of Addiction Medicine, Haukeland University Hospital; The Social Service of the Church: Kalfaret Treatment Center). Details regarding the substance abuse are shown in Table 2.

**Table 2.** Details regarding the substance abuse (*N* = 112).

	% of patients
Current preferred intoxicant	
Cannabis	30.4
Alcohol	22.3
Opiates	11.6
Amphetamine	11.6
Benzodiazepines	4.5
Other	2.7
No current use	4.5
Unknown	6.3
Time since initiation (years)	
0–5	19.6
6–10	30.4
11–15	21.4
≥ 16	11.6
Unknown	17.0
Age at initiation (years)	
≤ 12	9.8
13–16	48.2
17–20	14.3
21–39	9.0
Unknown	18.7

The comparison group consisted of patients from an outpatient teaching clinic at the Psychological Faculty, University of Bergen, Norway, diagnosed according to the *International Statistical Classification of Diseases and Related Health Problems 10th Revision* (ICD-10) (WHO, 1992) [F30–39, mood disorders (*n* = 38); F40–49, neurotic, stress-related, and somatoform disorders (*n* = 57); F60–69, disorders of adult personality and behaviour (*n* = 1); F90–98, behavioural and emotional disorders with onset usually occurring in childhood and adolescence (*n* = 1); and F99, unspecified mental disorder (*n* = 15)].

All participants gave their informed consent to participate in the study. Inclusion criteria were age > 16 years, and the ability to understand written and spoken Norwegian. Exclusion criteria were inadequate language skills, and intoxication related to substance use or active psychotic symptoms at the time of assessment (normally 1–2 weeks into treatment). The project was approved by the Regional Committee for Medical and Health Research Ethics (2009/1133) and the Norwegian Social Science Data Services (NSD).

### 2.2. Measurement

CMT was measured by the retrospective self-report questionnaire CTQ-SF (Bernstein & Fink, 1998), comprising 28 items formulated as behaviour-specific statements scored by respondents on a five-point Likert-type scale. Five items survey each of the five subscales: emotional abuse, physical abuse, sexual abuse, emotional neglect, and physical neglect. Scores are summarized into five subscale scores (range 5–25) and a total score (range 25–125). The Minimization–Denial subscale consists of three items, originally designed to assess a positive response bias (Bernstein et al., 2003). This scale will not be further discussed in this text, as it is rarely reported on in research literature, it is difficult to corroborate, and its value as a response bias index has been questioned (MacDonald, Thomas, MacDonald, & Sciolla, 2015). The Norwegian version of the CTQ-SF (Winje, Dovran, & Murison, 2003) has been found to have acceptable psychometric properties and reliability across different high-risk groups (Dovran et al., 2013). The overall internal reliability for the present sample was Cronbach’s *α* = .79. Subscale Cronbach’s *α* values were: emotional abuse *α* = .84, physical abuse *α* = .77, sexual abuse *α* = .94, emotional neglect *α* = .90, and physical neglect *α* = .62.

General psychological distress was measured by the SCL-90-R (Derogatis, 2009). The SCL-90-R is a self-report measurement consisting of 90 statements scored on a five-point Likert-type scale, measuring the occurrence of various symptoms during the past week. The Global Severity Index (GSI) is designed to measure overall psychological distress, and is recommended to be used as a summary of the test (Derogatis, 2009). In the present sample, the reliability for the SCL-90-R proved to be high, with Cronbach’s *α* = .97.

Post-traumatic stress symptoms were measured using the Impact of Event Scale – Revised (IES-R) (Weiss, ; Weiss & Marmar, 1997), a self-report measure consisting of 22 statements measuring three subscales related to PTSD: intrusion, avoidance, and hyperarousal. The 22 statements are scored on a five-point Likert-type scale, measuring the occurrence of symptoms during the past week. Even though the IES-R was not designed to make a categorical diagnosis of PTSD, it is common to quote cut-off scores in the literature. In this study, a relatively conservative cut-off sum score of 33 was applied, which is the recommended cut-off for probable post-traumatic stress symptoms severity levels in international (Creamer, Bell, & Failla, 2003) as well as in Norwegian samples of the general population (Heir, Piatigorsky, & Weisæth, 2009; Theodorescu, Hier, Hauff, Wentzel-Larsen, & Lien, 2012). The overall reliability for the IES-R was Cronbach’s *α* = .95, with subscale Cronbach’s *α* values as follows: hyperarousal *α* = .85, avoidance *α* = .88, and intrusion *α* = .91.

### 2.3. Procedure

Both groups completed a clinical test battery including the Norwegian versions of the CTQ-SF (Winje et al., 2003), SCL-90-R (Derogatis, 2009), and IES-R (Winje & Tungodden, 2001) at treatment initiation (normally 1–2 weeks into treatment). Administration of the IES-R requires that the informant experiences symptoms related to one or more traumatic event, and 98 participants (87.5%) in the SUD group and 82 participants (73.2%) in the comparison group responded.

### 2.4. Statistical analysis

The participants in the two groups were matched on gender and age.

Since the scores on the CTQ-SF were not normally distributed, the Mann–Whitney *U*-test was used for continuous variables and the Pearson chi-squared test was used for categorical variables. Pearson’s correlation coefficient, *r*, was used to calculate effect size. Cohen’s criteria were applied to the effect sizes small (*r = *.10), medium (*r *= .30), and large (*r *= .50).

Threshold scores from the CTQ-SF manual (Bernstein & Fink, 1998) were used to categorize the scores into none, low, moderate, or severe abuse and neglect. Since experiences of lower levels of CMT may be relatively common in the general population (Baker & Maiorino, 2010), a dichotomous variable separating ‘none’ and ‘low’ scores from ‘moderate’ and ‘severe’ was applied as a cut-off to determine caseness. Personal subscale means were imputed if CTQ-SF data were missing, and more than two missing items resulted in pairwise exclusion from the study. Six participants (2.7%) had one missing item. The cut-off level for clinical caseness of general psychological distress was an SCL-90-R GSI total-score ≥ 65 (male: ≥ 0.74; female: ≥ 0.94) (Derogatis, 2009). Caseness for post-traumatic stress symptoms was an IES-R sum score ≥ 33 (Creamer et al., 2003; Heir et al., 2009; Theodorescu et al., 2012).

A correlational analysis explored correlating scores on the different subscales of the CTQ-SF, CTQ-SF sum scores, IES-R, and SCL-90-R. A logistic regression analysis was conducted to investigate the odds ratios (ORs) for the different variables affecting the SCL-90-R GSI and the IES-R caseness.

A *p*-value of < .05 was considered statistically significant for all analyses. All data were analysed with STATISTICA version 13 (StatSoft, 2015).

## 3. Results

### 3.1. Demographic characteristics

Mean age was 27.5 (*SD* = 8.3) years in both groups. More patients in the comparison group had completed college or higher education compared to the SUD group (*χ*^2^ = 32.12, *p* < .001). Compared to the SUD group, more patients in the comparison group were studying or working (*χ*^2^ = 40.00, *p* < .001). In the SUD group, 49.1% were neither working nor studying, compared to only 10.7% in the comparison group. More patients in the comparison group were married or living with a partner compared to patients in the SUD group (*χ*^2^ = 9.31, *p *= .002). Almost all patients reported satisfactory housing conditions (Table 1).

### 3.2. Clinical characteristics

There were no differences in symptoms of general psychological distress (SCL-90-R GSI) or post-traumatic stress symptoms (IES-R) between genders in the total sample, but within the SUD group more males exceeded the cut-off for clinical caseness of general psychological distress (*χ*^2^ = 3.97, *p *= .046).

The SUD group had higher GSI scores on the SCL-90-R than the comparison group (*U *= 5315.0, *p* = .049). No differences were found between the groups in number of scores above the clinical cut-off on the SCL-90-R, or on post-traumatic stress symptoms, as measured by the IES-R (Table 3). An exploratory analysis did, however, reveal that the SUD group had higher scores on avoidance symptoms than the comparison group (*U* = 3196.0, *p *= .018, *r *= .18). No differences were found for the other subscales.

**Table 3.** Comparison of CTQ-SF, SCL-90-R, and IES-R sum scores, mean scores, subscale scores, and caseness by group.

	SUD group		Comparison group		Mann–Whitney *U*		Effect size	Caseness^b^			
Measure	*M (SD)*	*Median*	*M* (*SD*)	*Median*	*U*	*p*	Pearson’s *r*^a^	SUD group^c^	Comparison group^c^	*χ*^2d^	*p*
CTQ-SF sum	46.94 (15.49)	43.00	37.22 (11.59)	34.00	3685.5	.000*	.36				
CTQ-SF mean	1.88 (0.62)	1.72	1.49 (0.46)	1.36	3685.5	.000*	.36				
Emotional abuse	11.04 (4.66)	10.00	8.54 (4.49)	7.00	4038.0	.000*	.31	41 (36.61%)	17 (15.18%)	13.401	.000*
Physical abuse	7.22 (3.38)	6.00	5.85 (2.16)	5.00	4449.0	.000*	.25	21 (18.75%)	6 (5.36%)	9.475	.002*
Sexual abuse	7.83 (5.17)	5.00	5.56 (1.63)	5.00	4824.0	.003*	.20	32 (28.57%)	11 (9.82%)	12.692	.000*
Emotional neglect	12.69 (4.92)	12.00	10.74 (4.41)	10.00	4899.5	.005*	.19	37 (33.04%)	27 (24.11%)	2.188	.139
Physical neglect	8.16 (2.96)	8.00	6.54 (2.51)	5.00	4016.0	.000*	.31	33 (29.46%)	14 (12.5%)	9.720	.002*
SCL-90-R GSI	1.20 (0.72)	1.10	0.99 (0.52)	0.88	5315.0	.049*	.14	76 (67.86%)	66 (58.93%)	1.924	.165
IES-R sum	32.98 (20.21)	29.50	26.83 (19.97)	26.00	3341.0	.052	.15	43 (38.39%)	33 (29.46%)	0.179	.673

CTQ-SF, Childhood Trauma Questionnaire – Short Form; SCL-90-R GSI, Symptom Checklist-90 – Revised Global Severity Index; IES-R, Impact of Event Scale – Revised.

^a^Cohen’s criteria: small (*r* = .10), medium (*r* = .30), large (*r* = .50). ^b^*n* (%) in each group that reported subscale scores in the moderate–severe range on the CTQ-SF subscales, scored above clinical caseness on the SCL-90-R GSI [T-score > 63 ≤ GSI raw score 0.74 (male) and 0.94 (female)] or on the IES-R (sum score ≥ 33). ^c^*n* = 112 for all measures, except for the IES-R where *n *= 98 in the SUD group and *n *= 82 in the comparison group. ^d^*df *= 1.

*Significant at *p *< .05 (two-tailed).

### 3.3. Group differences on CTQ-SF

The SUD group had higher CTQ-SF sum scores than the comparison group (*U *= 3685.5, *p < *.001, *r *= .36), and scored higher on all five subscales (Table 3).

More patients from the SUD group than the comparison group exceeded the cut-off for moderate to severe levels of emotional abuse (*χ*^2^ = 13.40, *p *< .001), physical abuse (*χ*^2^ = 9.48, *p *= .002), sexual abuse (*χ*^2^ = 12.69, *p *< .001), and physical neglect (*χ*^2^ = 9.72, *p *= .002). No differences were found for emotional neglect caseness (Table 3).

### 3.4. Gender differences on CTQ-SF

Analysis of the total sample revealed that more females than males scored above the cut-off for moderate to severe levels of emotional abuse (*χ*^2^ = 7.03, *p *= .008), physical abuse (*χ*^2^ = 4.61, *p *= .032), and sexual abuse (*χ*^2^ = 4.48, *p *= .034).

In the SUD group, more females than males exceeded the cut-off for moderate to severe levels of emotional abuse (*χ*^2^ = 5.75, *p *= .017), physical abuse (*χ*^2^ = 5.52, *p *= .019), and sexual abuse (*χ*^2^ = 6.40, *p *= .011). Females in the SUD group had higher scores on emotional abuse (*U *= 1136.0, *p *= .017) and sexual abuse (*U *= 1136.5, *p *= .017) and on the CTQ-SF sum score (*U *= 1155.0, *p *= .023) (Table 4).

**Table 4.** Comparison of CTQ-SF sum scores, subscale scores, and caseness by gender in the SUD group.

	Males		Females		Mann–Whitney *U*		Effect size	Caseness^b^			
Measure	*M* (*SD*)	*Mdn*	*M (SD)*	*Mdn*	*U*	*p*	Pearson’s *r*^a^	Males^c^	Females^c^	*χ*^2d^	*p*
CTQ-SF sum	43.50 (12.26)	40	51.37 (18.03)	46	1155.0	.023*	.22				
CTQ-SF mean	1.74 (0.49)	1.06	2.05 (0.72)	1.84	1155.0	.023*	.22				
Emotional abuse	10.12 (4.43)	9	12.22 (4.74)	11	1136.0	.017*	.23	17 (26.98%)	24 (48.98%)	5.746	.017*
Physical abuse	6.68 (2.61)	5	7.92 (4.09)	6	1352.5	.264	.11	7 (11.11%)	14 (28.57%)	5.516	.019*
Sexual abuse	6.43 (3.14)	5	9.63 (6.58)	5	1136.5	.017*	.23	12 (19.05%)	20 (40.82%)	6.4	.011*
Emotional neglect	12.42 (4.95)	12	13.02 (4.91)	12	1444.5	.563	.05	18 (28.57%)	19 (38.78%)	1.297	.255
Physical neglect	7.84 (2.73)	7	8.57 (3.22)	8	1339.5	.227	.11	16 (25.40%)	17 (34.69%)	1.146	.284
SCL-90-R GSI	1.09 (0.66)	0.95	1.11 (0.60)	0.97	5876.5	.537	.04	87 (69.10%)	55 (56.12%)	3.97	.046*
IES-R sum	28.57	26.67	32.00 (19.39)	30.00	3584.5	.120	.10	37 (29.37%)	39 (39.80%)	1.17	.279

CTQ-SF, Childhood Trauma Questionnaire – Short Form; SUD, substance use disorder; SCL-90-R GSI, Symptom Checklist-90 – Revised Global Severity Index; IES-R, Impact of Event Scale – Revised.

^a^Cohen’s criteria: small (*r* = .10), medium (*r* = .30), large (*r* = .50). ^b^*n* (%) in each group that reported subscale scores in the moderate–severe range. ^c^*n* = 112  for all measures, except for the IES-R where *n* = 98. ^d^*df *= 1.

*Significant at *p *< .05 (two-tailed).

Within the comparison group, males had higher scores on emotional neglect (*U *= 1191.5, *p *= .039). Further analysis revealed no other gender differences within this group.

### 3.5. Intercorrelations

For females, all variables on the CTQ-SF correlated except for sexual abuse, which did not correlate with physical neglect, SCL-90-R scores, and IES-R scores. Males showed a similar pattern; all CTQ-SF subscales correlated except for sexual abuse. However, physical abuse did not correlate with the SCL-90-R, and neither physical abuse, nor sexual abuse, nor emotional neglect correlated with the IES-R sum (Table 5).

### 3.6. Odds ratios

The parameter estimates for SCL-90-R caseness together with standard errors (*SE*s) and confidence intervals (CIs) by the logistic regression model are shown in Table 6. The 95% CIs for ORs of the effect of the different parameters show that gender (OR = .381, *p *= .010), civil status (OR = 2.313, *p* = .042), and emotional neglect (OR = 2.809, *p* = .038) were significant (model *χ*^2^ = 27.48, pseudo *R*^2^ = .125, *n* = 222).

**Table 5.** Intercorrelations, means, and standard deviations for males and females in both groups.

		1	2	3	4	5	6	7	8	*M*	*SD*
1	Emotional abuse	–	.65*	.33*	.61*	.54*	.81*	.38*	.31*	10.69	5.04
2	Physical abuse	.49*	–	.42*	.54*	.63*	.81*	.30*	.40*	6.99	3.63
3	Sexual abuse	.15	.17	–	.43*	.20	.65*	.10	.18	7.70	5.15
4	Emotional neglect	.53*	.30*	.18	–	.71*	.85*	.37*	.22*	11.69	4.89
5	Physical neglect	.62*	.51*	.19	.59*	–	.75*	.29*	.23*	7.48	3.12
6	CTQ-SF sum	.84*	.65*	.40*	.80*	.82*	–	.37*	.34*	44.55	16.89
7	SCL-90-R sum	.32*	.16	.28*	.28*	.25*	.36*	–	.51*	102.61	56.05
8	IES-R sumq	.29*	.11	.13	.14	.29*	.27*	.49*	–	32.00	19.39
	*M*	9.55	6.45	6.01	12.18	7.50	41.69	102.53	28.58		
	*SD*	4.61	2.64	32.43	4.63	2.81	12.53	60.96	21.00		

CTQ-SF, Childhood Trauma Questionnaire – Short Form; SCL-90-R, Symptom Checklist-90 – Revised; IES-R, Impact of Event Scale – Revised.

*Significant at p < .05. *n* = 98 for females, *n* = 126 for males. Females are presented above the diagonal, males are presented below the diagonal.

**Table 6.** Logistic regression model for SCL-90-R GSI caseness.

Parameter	OR	*SE*	95% CI		*Z*-value	*p* (> *Z*)
			Lower bound	Upper bound		
Group	1.100	0.460	0.485	2.498	.23	.820
Female	0.381	0.143	0.183	0.796	−2.57	.010*
Work	0.446	0.184	0.198	1.003	−1.95	.051
Civic	2.313	0.956	1.029	5.199	2.03	.042*
Emotional abuse	2.384	1.296	0.821	6.920	1.60	.110
Physical abuse	1.185	0.962	0.241	5.822	0.21	.834
Sexual abuse	1.457	0.781	0.510	4.165	0.70	.482
Emotional neglect	2.809	1.402	1.056	7.470	2.07	.038*
Physical neglect	1.020	0.581	0.334	3.112	0.03	.972

SCL-90-R GSI, Symptom Checklist-90 – Revised Global Severity Index; OR, odds ratio; CI, confidence interval.

Model *χ*^2^ = 27.48, *p* < .01; pseudo *R*^2^ = .12. *n* = 222.

*Significant at *p* < .05.

**Table 7.** Number of childhood maltreatment trauma events by group and gender (%).

	0	1	2– 3	4–5
Total sample^a^	50.5	21.4	18.8	9.4
SUD group^b^	35.7	25.0	24.1	15.2
Females^c^	26.5	20.4	28.6	24.5
Males^d^	42.9	28.6	20.6	7.9
Comparison group^b^	65.2	17.9	13.4	3.6
Females^c^	63.3	16.3	18.4	2.0
Males^d^	66.7	19.0	9.5	4.8

SUD, substance use disorder.

^a^*n *= 224, ^b^*n *= 112, ^c^*n* = 49, ^d^*n *= 63.

In a logistic regression model of the IES-R sum caseness, work status (OR = .399, *p* = .041) and civil status (OR = 2.825, *p* = .021) were significant within the 95% CI for the OR of the effect of the different parameters (model *χ*^2^ = 13.72, pseudo *R*^2^ = .091, *n* = 178).

### 3.7. Prevalence of multiple traumas

In the total sample, 49.6% reported experiences of moderate to severe CMT, and the majority of these had experienced multiple traumas. More patients in the SUD group had experienced multiple CMTs at moderate to severe levels (*U = *4205.0, *p *< .001). No gender differences were found when including both groups, or in the comparison group. Within the SUD group, more females than males had experienced two to five different moderate to severe CMTs (*U* = 1105.5, *p* = .010) (Table 7).

## 4. Discussion

The occurrence of CMT proved high in both patient groups. Approximately half of the sample reported moderate to severe CMT, and the majority of these reported multiple traumas. The results add further support to the association between CMT and adult mental health problems in general, and suggest a particularly strong association for adults suffering from SUD.

Our hypothesis that the occurrence of CMT would be higher in the SUD group than the comparison group was confirmed, as the patients in treatment for SUD had higher scores on all five CTQ-SF subscales. More patients from the SUD group reported moderate to severe childhood emotional, physical, and sexual abuse, as well as physical neglect. The occurrence of moderate to severe emotional neglect did not differ between the groups, but the SUD patients reported higher sum scores. Moderate to severe emotional neglect appeared to be common in both patient groups.

Analyses revealed gender differences confined to the SUD group, where more females reported moderate to severe emotional, physical, and sexual abuse compared to males. Compared to the comparison group, reports of moderate to severe emotional abuse were nearly doubled for males and more than tripled for females in the SUD group; moderate to severe physical abuse was twice as common among males and more than five times as common among female SUD patients; and moderate to severe sexual abuse was twice as common among males and four times as common among females in the SUD group.

Our hypothesis that CMT was associated with current post-traumatic stress symptoms and general psychological distress was confirmed within the SUD group. CMT was not associated with post-traumatic stress symptoms in the comparison group, and was only associated with symptoms of general psychological distress for females in this group. These differences could be related to differences in severity and multiplicity of experienced CMT, with almost no associations in the comparison group as opposed to the more heavily traumatized SUD group. Previous research has found an association between avoidance strategies and the promotion and maintenance of psychopathology (Hayes, Wilson, Gifford, Follette, & Strosahl, 1996; Min, Farkas, Minnes, & Singer, 2007), and the higher scores on avoidance symptoms among the patients in the SUD group could be interpreted as a cognitive style predisposing these patients to both post-traumatic stress symptoms and SUD.

Research has consistently found that females are more vulnerable to the development of PTSD than males (Breslau, 2009; Tolin & Foa, 2006). The present study found no gender differences in clinical post-traumatic stress symptoms. However, while only emotional abuse and physical neglect correlated with post-traumatic stress symptoms for males, all different types of CMT except sexual abuse correlated with post-traumatic stress symptoms for the females.

In line with previous research indicating that sexual abuse is perpetuated by an acquaintance or relative other than the parent (Gilbert et al., 2009), sexual abuse tended to co-occur with other trauma to a lesser degree than other forms of CMT in the present study. Thus, a positive relation to one or more adult caregivers may function as a protective factor when a child is exposed to sexual abuse. Childhood sexual abuse, however, correlated with the CTQ-SF sum score, which again correlated with current post-traumatic stress symptoms and general psychological distress for both genders. Previous research has found that while the association between childhood sexual abuse and PTSD sometimes appears to be straightforward, it is often mediated by a broader range of trauma exposure, particularly other CMT or adult sexual assault (Banyard, Williams, & Siegel, 2001). Other research indicates that more serious sexual abuse increased the risk for later mental health problems even after adjusting for confounding variables (Fergusson, Boden, & Horwood, 2008). In line with previous research (Fergusson, McLeod, & Horwood, 2013), the results from the present study may suggest that childhood sexual abuse to a greater degree is associated with adult psychopathology in cases of more severe sexual abuse, or in cases in which other forms of CMT are present.

In accordance with previous research (Dong et al., 2004; Dube et al., 2003; Green et al., 2010; Kessler et al., 2010), the majority of patients in the present study reporting any CMT had experienced multiple traumas. Multiple CMTs were more often reported by the SUD group, particularly by females. More than 50% of females in the SUD group reported two to five different moderate to severe CMTs, and approximately 25% reported four or five different traumas. This finding is in line with previous research illustrating the traumatic load experienced by substance abusers, females in particular (Daigre et al., 2015).

Results from the regression analysis indicated that the model explained 12% of the variance in SCL-90-R caseness and 9% of the variance in IES-R caseness. These results may indicate that other parameters than those included in the model contributed to the variance. The strong intercorrelations among the different variables of the CTQ-SF may, however, affect the results, and a better approach might have been to control for the CTQ-SF total caseness scores.

The SUD group reported a higher level of general psychological distress than the comparison group, but no differences in number of scores above the clinical cut-off. The measures of post-traumatic stress symptoms revealed a non-significant trend of higher sum scores as well as number of patients above the cut-off for clinical caseness in the SUD group. It is worth noting that Rash, Coffey, Baschnagel, Drobes, and Saladin (2008) found that the cut-off value of 33, as suggested by Creamer et al. (2003), was too conservative for capturing patients with PTSD in a sample of substance abusers and recommended a cut-off value of 22 to be applied for SUD samples. It is possible that the current estimate of patients above the cut-off for clinical post-traumatic stress symptoms is too low, but a lower cut-off would increase the probability of a type I error.

Estimated clinical caseness for post-traumatic stress symptoms was high for both groups compared to estimates of lifetime PTSD prevalence of 5–10% in the general population (Ozer, Best, Lipsey, & Weiss, 2008). Although many patients in both groups scored above the cut-off for clinical post-traumatic stress symptoms, the reported symptoms only correlated with the sum of CMT for the SUD group, suggesting a stronger and possibly more direct association between CMT and post-traumatic stress symptoms for these patients. The present study searched for current post-traumatic stress symptoms only, leaving the question of group differences in lifetime prevalence of post-traumatic stress symptoms open.

### 4.1. Limitations

Several limitations can be noted for the present study. First, the method of retrospective self-report could lack validity, as self-reports may be influenced by forgetting, lack of awareness, or reporting bias related to emotional states or gender roles (Tolin & Foa, 2006). However, false negatives appear to be more common than false positives in the general population, and possible biased associations between retrospective reports of CMT and psychopathology may be the result of the rehearsal of memories, rather than bias related to current emotional states (Hardt & Rutter, 2004).

A second limitation is that details regarding the CMT, such as age at onset, duration, and relationship to the perpetrator, are not accounted for. These aspects may be important confounding variables impacting outcomes related to the occurrence and severity of adult psychopathology, SUD, or both (Tolin & Foa, 2006).

All associations found in the current study showed small to medium effects; however, the clinical implications are likely to be relevant as the results are highly significant and the samples are relatively small. Although there is a strong association between CMT and psychopathology and SUD in the current study, it is important to notice that many subjects did not report such experiences, or could not discern whether their present symptoms were related to childhood maltreatment or other childhood trauma. The reasons behind adult psychopathology and SUD are complex and diverse. However, the present study adds further support to consider a strong association between experiences of CMT, adult psychopathology, and substance abuse and dependence in treatment intervention plans.

## 5. Conclusions

The present study found that approximately half of the adult patients had experienced moderate to severe CMT. The patients in treatment for SUD reported having experienced more severe and multiple CMTs than patients in treatment for mild to moderate psychopathology. Females with SUD reported more severe and multiple CMTs compared to males in the same group. The demographic data from the present study indicate worse life outcomes for patients with SUD compared to mental health patients without SUD. Both groups had high scores on clinical post-traumatic stress symptoms.

Based on these findings, we recommend that clinicians systematically screen for CMT. Screening for post-traumatic stress symptoms should be conducted routinely with SUD patients, as previous research has indicated poorer outcomes of SUD treatments when PTSD is left untreated (Read, Brown, & Kahler, 2004). Avoidant coping style may have an important mediating effect in the association between CMT and SUD, and may support the self-medication hypothesis (Khantzian, 1985).
